# Association Between Prior NRICM101 Use and Response to Incentive Spirometer Training in Patients with Long COVID

**DOI:** 10.3390/healthcare14121630

**Published:** 2026-06-09

**Authors:** Yao-Hsiang Chen, Chia-Huei Lin, Ju-Han Liu, Hsin-An Lin, Yu-Shan Hsieh

**Affiliations:** 1 School of Nursing, National Taipei University of Nursing and Health Sciences, No. 365, Mingde Rd., Beitou Dist., Taipei City 11230, Taiwan; 2 Department of Nursing, Tri-Service General Hospital Songshan Branch, Taipei City 10581, Taiwan; 3 School of Nursing, National Defense Medical University, Taipei City 11420, Taiwan; 4 Graduate Institute of Medical Sciences, National Defense Medical University, Taipei City 11420, Taiwan; 5 Institute of Traditional Medicine, School of Medicine, National Yang Ming Chiao Tung University, Taipei City 11230, Taiwan; 6 Division of Infection, Department of Medicine, Tri-Service General Hospital Songshan Branch, National Defense Medical Center, Taipei City 10581, Taiwan; 7 Performance Examination Center, National Taipei University of Nursing and Health Sciences, Taipei City 11230, Taiwan

**Keywords:** NRICM101, long COVID, incentive spirometer, dyspnea

## Abstract

**Background**: Inspiratory training using an incentive spirometer (IS) improves respiratory symptoms and functional status in Long COVID. In Taiwan, NRICM101 is commonly used during the acute phase; however, its impact on rehabilitation outcomes remains unclear. **Objective**: This study evaluated the effects of IS-based respiratory training and the potential influence of prior NRICM101 use. **Methods**: Participants were grouped by time since recovery, and pre-post changes after six weeks of IS training were compared between those with and without self-reported prior NRICM101 use. Primary outcomes were dyspnea and functional status; secondary outcomes included six-minute walk distance and arterial oxygen content. **Results**: IS training significantly improved dyspnea (*p* < 0.0001), functional status (*p* < 0.0001), and exercise endurance (NRICM101 group: *p* = 0.002; no NRICM101 group: *p* < 0.0001). Stratified analysis showed that when initiated within three months of recovery, participants without prior NRICM101 use had greater improvements in dyspnea (*p* < 0.0001), functional status (*p* = 0.001), and exercise endurance (*p* < 0.0001). **Conclusions**: Prior NRICM101 use may be associated with different rehabilitation patterns, likely reflecting differences in recovery trajectory or patient behavior rather than purely pharmacological effects.

## 1. Introduction

### 1.1. Introduction to Dyspnea in Long COVID

Prior to the Coronavirus disease 2019 (COVID-19) pandemic, even in cases with a relatively short duration of infection, long-term sequelae had been observed following viral infections such as severe acute respiratory syndrome (SARS) and Middle East respiratory syndrome (MERS) [1]. The sequelae of severe acute respiratory syndrome coronavirus 2 (SARS-CoV-2) infection, known as long COVID, has become a significant global public health issue. Long COVID refers to symptoms that persist or newly emerge more than four weeks after acute COVID-19 infection, with some patients experiencing symptoms lasting over three months or even exceeding one year [2]. Long COVID is believed to involve chronic multisystem effects, with common symptoms including fatigue, dyspnea, cognitive impairment (brain fog), muscle and joint pain, palpitations, chest pain, anxiety, and sleep disturbances. Recent studies have also highlighted the potential role of neurological, cognitive, and autonomic manifestations in contributing to persistent functional impairment and rehabilitation needs in patients with long COVID [3,4]. These symptoms may persist or recur after initial recovery and often exhibit fluctuating or recurrent characteristics [5].

Among these symptoms, dyspnea is a key manifestation affecting quality of life and functional status and is often accompanied by decreased exercise tolerance and persistent chest tightness [6]. These respiratory symptoms may limit daily activities and work capacity and contribute to persistent physical and psychological burdens. Follow-up studies have shown that long-term symptoms may persist for more than one year after infection, highlighting the substantial long-term health burden associated with long COVID [7,8].

Current management of long COVID remains mainly supportive and symptom-based, and most recommendations advocate a multidisciplinary rehabilitation approach including respiratory training, physical therapy, psychological support, and cognitive rehabilitation [9]. Given the clinical significance of dyspnea in long COVID, the World Health Organization (WHO), European Respiratory Society (ERS), and American Thoracic Society (ATS) have all suggested that respiratory training may help alleviate breathing-related symptoms in affected patients [10].

Furthermore, long COVID-related services vary significantly across healthcare systems, and some regions continue to experience limitations in specialized care resources and rehabilitation accessibility [9]. Therefore, developing safe, feasible, and accessible rehabilitation strategies has important clinical and public health implications.

### 1.2. Incentive Spirometer-Based Respiratory Rehabilitation in Long COVID

The incentive spirometer (IS) is a common device for inspiratory training. This concept originated from observations of promoting postoperative lung expansion in the 1960s. An IS guides patients to perform slow and deep inspiratory exercises by simulating a sustained maximum inhalation [11]. Through visual feedback mechanisms, the device encourages maintenance of stable inspiratory flow and sufficient inspiratory volume, thereby promoting alveolar expansion and improving ventilation efficiency. Due to its ease of operation, low cost, and high safety, the IS has been widely used in the prevention of postoperative pulmonary complications and respiratory function rehabilitation [12].

In our previous research on COVID-19, the IS had also been used as a viable respiratory training tool [13]. Studies have shown that daily use of an IS for breathing exercises in outpatients within 30 days of diagnosis of COVID-19 can significantly increase maximum inspiratory capacity without observed worsening of symptoms [12]. Other related clinical trials have also suggested that IS intervention may improve oxygenation parameters (PaO_2_/FiO_2_), shorten hospital stays, and achieve symptom relief [14]. In addition, both respiratory rehabilitation programs incorporating the use of an IS and other respiratory training methods can improve lung function and dyspnea among patients with long COVID [15,16].

### 1.3. NRICM101 and Its Potential Role in COVID-19 Recovery

Taiwan Chingguan Yihau (NRICM101) is a traditional Chinese medicine compound developed by the National Research Institute of Chinese Medicine in Taiwan during the COVID-19 pandemic in 2020. NRICM101 contains the following ingredients: *Houttuynia cordata*, *Isatis indigotica root*, *Scutellaria baicalensis*, *Trichosanthes kirilowii fruit*, *Schizonepeta tenuifolia*, *Morus alba leaf*, *Mentha haplocalyx*, *Magnolia officinalis*, *Saposhnikovia divaricata*, and *Glycyrrhiza uralensis* (processed). These herbs are boiled in 1000 mL of water and concentrated to 300 mL. Since April 2020, this formula has been used as an adjunctive medication for the routine treatment of COVID-19 in Taiwan. This herbal formula was developed based on clinical symptoms, the pharmacological properties of the herbs, and lessons learned from the 2003 SARS outbreak. NRICM101 is primarily used for confirmed patients with COVID-19 who have mild symptoms but who are at high risk of severe illness. Pharmacological studies suggest that NRICM101 may exert antiviral and anti-inflammatory effects by modulating the host immune response and interfering with viral replication [17]. Some observational studies have also shown that the use of NRICM101 is associated with symptom recovery, improvement in chest imaging, increased seroconversion rates detected with reverse transcription polymerase chain reaction (RT-PCR), and a shorter hospitalization duration [18]. The Asia-Pacific Economic Cooperation (APEC) Health Working Group has included NRICM101 as a reference treatment option for traditional medicine interventions against COVID-19 in its evidence-based guidelines [19].

In the early stages of the pandemic when treatment guidelines were not yet clearly established, NRICM101, a traditional medicine developed domestically, enjoyed a high level of social and cultural acceptance in Taiwan, with public funding policies facilitating its widespread use. Tseng et al. conducted a nationwide online survey in 2023, which included 7042 participants, of whom 67.6% had previously used NRICM101 [20]. Clinical data analysis showed that the use of NRICM101 may be associated with intubation, admission to intensive care units, or reduced risk of death [18]. Another study indicated that its use was associated with shorter hospital stays and reduced mechanical ventilation time in critically ill patients, despite the fact that no significant decrease in in-hospital mortality was observed [21].

The pathological mechanism underlying long COVID is believed to be related to autoimmune dysregulation and persistent pathological inflammatory responses. Based on this mechanism of action, plant-derived bioactive compounds with antiviral, anti-inflammatory, and immunomodulatory properties may enhance the recovery of subsequent symptoms after the acute phase of COVID-19. Flavonoids are one of the key components of NRICM101 and have been reported to have anti-pulmonary fibrosis and lung-protective effects [22,23].

While preliminary recommendations have been made for rehabilitation strategies for long COVID based on the abovementioned literature review, particularly the potential benefits of respiratory training in improving dyspnea and functional status, the potential influence of different background factors on rehabilitation responses has not been explored. Moreover, research on NRICM101 has largely focused on its anti-inflammatory and immunomodulatory effects in the acute phase of COVID-19, and the relevant empirical evidence remains quite limited on whether it may affect the subsequent rehabilitation outcomes of patients with long COVID. In particular, whether prior NRICM101 use may be associated with different responses to respiratory rehabilitation has not been well explored. Therefore, we aim to investigate the improvement in dyspnea and functional status among patients with long COVID who have used NRICM101 following breathing exercises using an IS, and to further evaluate the possible association between the use of NRICM101 and respiratory rehabilitation outcomes.

## 2. Materials and Methods

### 2.1. Study Design and Participants

This study was designed as a quasi-experimental study. Overall, 49 eligible participants were enrolled in this study at a regional teaching hospital in Taipei, Taiwan, from 1 July 2023 to 11 May 2024. This study aims to evaluate the effectiveness of IS training on dyspnea and functional status in patients with long COVID, and the association between prior NRICM101 use and rehabilitation outcomes.

Participants were first randomized to either the intervention group or the control group. Participants in the intervention group were subsequently stratified according to the duration since recovery from COVID-19 (<3 months and 3–12 months after recovery), forming EG1 and EG2, respectively. Outcome changes before and after IS training were then compared across groups to explore the association between NRICM101 use and pulmonary rehabilitation outcomes.

The study groups were defined as follows: (1) control group (CG): participants who did not receive the IS intervention; (2) experimental group 1 (EG1): those who received the IS intervention within 3 months after COVID-19 recovery; (3) experimental group 2 (EG2): participants who received the IS intervention between 3 and 12 months after COVID-19 recovery.

### 2.2. Inclusion and Exclusion Criteria of Participants

Participants were adults diagnosed with COVID-19 within the past year, who completed acute-phase treatment and recovered, and who continued to experience at least one respiratory-related long COVID symptom. COVID-19 diagnosis was confirmed with supporting medical documentation (e.g., medical records or test reports) using ICD-10 diagnostic codes U07.1 (confirmed COVID-19 infection) or U09.0 (post COVID-19 condition, unspecified).

Inclusion Criteria: (1) Individuals who recovered from COVID-19 within the past year (confirmed by a negative rapid antigen test). The corresponding ICD-10 codes included U07.1 (COVID-19; virus identified) and U09.0 (Post COVID-19 condition; unspecified). (2) Presence of at least one respiratory-related symptom related to Long COVID (such as exertional dyspnea, shortness of breath, chest tightness, cough, or dyspnea). (3) Aged between 20 and 90 years. (4) Intact consciousness with normal cognitive function and behavioral stability. (5) Ability to communicate, verbally or non-verbally, and understand Mandarin Chinese or a Taiwanese dialect. (6) Willingness to participate in the study and complete study procedures.

Exclusion Criteria: (1) Individuals with severe disabilities or those who are permanently bedridden. (2) Diagnosed with dementia (such as Alzheimer’s disease and Parkinson’s disease). (3) Presence of acute psychiatric symptoms that impair communication. (4) Individuals with a high risk of litigation. (5) Diagnosed with chronic obstructive pulmonary disease (COPD) or any other respiratory disorders. (6) Diagnosed with moderate to severe heart disease.

### 2.3. Intervention

In Taiwan, the recommended clinical regimen for NRICM101 is to initiate treatment within 5 days of symptom onset, with a dose of 100 mL administered thrice daily, 30 min after meals, for a total duration of 5 days. NRICM101 is developed by the National Research Institute of Chinese Medicine (NRICM) and manufactured by eight licensed pharmaceutical companies according to standardized production protocols to ensure batch-to-batch consistency of active components and quality. The preparation is performed by authorized manufacturers using fixed proportions, with a full set of herbal ingredients decocted in 1 L of water and subsequently concentrated to 300 mL through boiling and simmering. This standardized process, rather than patient-prepared decoction, ensures consistency in dosage and composition and minimizes variability in drug concentration [17].

Prior NRICM101 exposure was assessed by participant self-report and generally followed the standardized regimen described above. Because NRICM101 was commonly obtained through herbal medicine stores during the COVID-19 pandemic in Taiwan, detailed information regarding dosage, frequency, and source of acquisition was not consistently available through hospital medical records. Therefore, prior NRICM101 exposure could not be independently verified and was classified based on participant report. Information regarding NRICM101 use was self-reported by participants at study entry and was not considered an interventional variable. Only the experimental groups received IS-based respiratory training, while the CG received usual care without additional pulmonary rehabilitation interventions and continued their usual daily activities during the study period. All participants underwent outcome assessments at baseline and at 6 weeks post-intervention to evaluate changes in dyspnea and functional status.

Participants in the EG received inspiratory training using an IS (Taiwan FDA Device Approval No. 003427. Besmed Health Business Corp., New Taipei City, Taiwan). They were instructed to complete three training sessions per week, each consisting of 30 sustained inhalations of at least 3 s to promote lung expansion and inspiratory muscle endurance. The training intensity was individually adjusted based on the pulmonary function and tolerance of participants to ensure safety and feasibility.

The initial IS training session was supervised by a nurse, who provided instructions and guidance to ensure participants fully understood the standardized training protocol. Written informed consent was obtained from all participants before enrollment. Subsequent sessions were performed independently by participants at home. To support adherence and evaluate intervention feasibility, the research team conducted regular telephone follow-ups at week 3. Post-intervention assessments were conducted after completion of the 6-week training program.

### 2.4. Outcome Measures

All participants were assessed at baseline (pre-intervention) and 6 weeks post-intervention. Analyses were conducted according to prior NRICM101 use to compare changes in respiratory function and functional status. The primary outcomes included subjective dyspnea and functional status, while the secondary outcomes comprised exercise capacity and pulmonary oxygenation indices.

Subjective dyspnea was assessed using the Dyspnoea-12 (D-12) scale, which comprises 12 items covering physical and affective components of breathlessness. Higher total scores indicate greater severity of dyspnea. The Chinese version of the D-12 demonstrates good reliability and validity [24,25,26].

Functional status was evaluated using the Chinese version of the Post-COVID-19 Functional Status (PCFS) scale. The PCFS scale is graded from 0 to 4 (0 = no functional limitation; 1–4 = increasing levels of functional limitation), with grade 5 indicating death. It is used to assess the impact of COVID-19 on daily activities following recovery and has been validated as an effective measure of functional impairment in patients with long COVID [27,28,29].

Exercise capacity and cardiopulmonary function were assessed using the Six-Minute Walk Test (6MWT). The primary outcome was the six-minute walk distance (6MWD), with heart rate and oxygen saturation recorded before and after the test to evaluate exercise tolerance and oxygenation response [30].

For oxygenation-related parameters, because the present study involved clinically stable community-dwelling participants, pulse oximetry-derived SpO_2_ was used as a non-invasive surrogate of SaO_2_ for CaO_2_ estimation, consistent with previous literature [31,32,33]. Arterial oxygen content (CaO_2_) was estimated using the formula CaO_2_ = Hb × SaO_2_ × 1.34 ÷ 100 (mL/dL) and was used as an exploratory oxygenation-related parameter rather than a direct measure of pulmonary oxygenation function [32,34].

### 2.5. Statistical Analyses

All data were organized using Excel 2019 (Microsoft Corp., Redmond, WA, USA) and analyzed with SPSS version 18.0 (SPSS Inc., Chicago, IL, USA). Descriptive statistics were reported as means, standard deviations, and percentages. Inferential analyses included independent and paired *t*-tests, ANOVA, chi-square tests, and both linear and logistic regression. Generalized Estimating Equations (GEEs) were used to evaluate group-by-time interaction effects and assess the efficacy of the intervention. The GEE models included group, time, and group-by-time interaction terms, and an autoregressive correlation structure (AR1) was applied. A *p*-value of <0.05 was considered statistically significant.

### 2.6. Ethical Approval and Informed Consent

This study was approved on 12 May 2023 by the Ethics Committee of the Tri-Service General Hospital (A202305044), and all participants were informed of the details of the trial and signed informed consent forms before the experiment. This study was conducted in accordance with the Declaration of Helsinki.

## 3. Results

**Figure 1:**
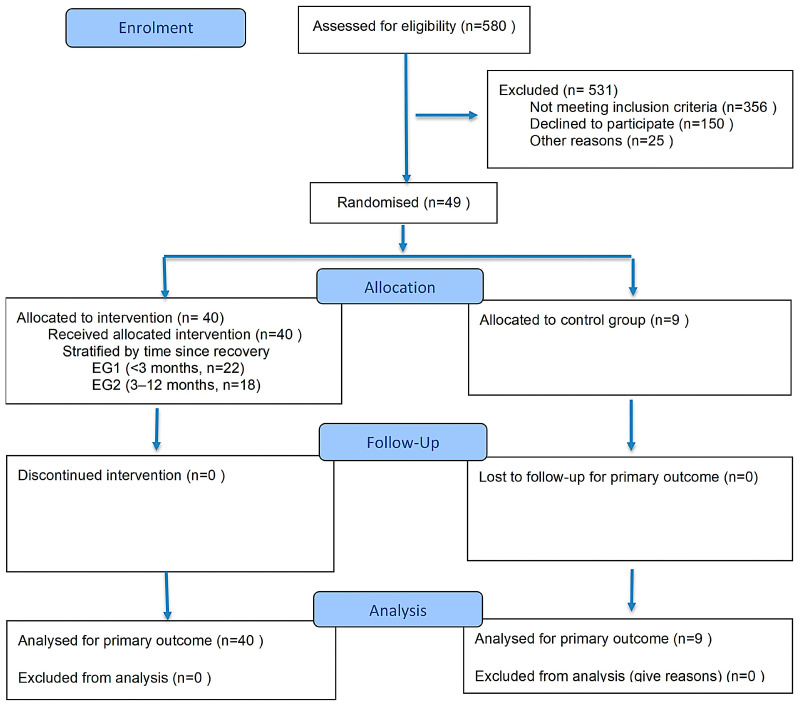
Flow diagram of participant enrollment, group allocation, follow-up, and analysis.

This study included a total of 49 participants; of these, 26 had not used NRICM101, and 23 had used the formula. Among all participants, the mean age was 39.5 years (median 34 years); 59.2% were female, and 40.8% were male. Participants were divided into the three main groups including the control group (CG), experimental group 1 (EG1) (within 3 months after recovery), and experimental group 2 (EG2) (3–12 months after recovery) (Figure 1). They were further stratified based on whether NRICM101 was administered. There was no significant difference in gender distribution (*p* = 0.461) or age (*p* = 0.88) across the groups. There was also no statistically significant difference in height (*p* = 0.319) or body weight (*p* = 0.136) between groups of participants. In terms of comorbidities, the prevalence of hypertension, diabetes, and smoking did not differ significantly among groups (*p* > 0.05), whereas the distribution of cardiovascular diseases showed a statistically significant difference (*p* = 0.006). In addition, there were no statistically significant differences in the number of infections (*p* = 0.148) and exercise habits (*p* = 0.258) between the groups.

In terms of baseline indicators related to function and respiration, no significant differences in Post-COVID-19 Functional Status (PCFS) scale scores (*p* = 0.235), Dyspnoea-12 (D-12) scores (*p* = 0.604), six-minute walk distance (6MWD) test results (*p* = 0.572), heme concentration (*p* = 0.122), hematocrit (*p* = 0.115), peripheral oxygen saturation (SpO_2_) (*p* = 0.204), and arterial oxygen content (CaO_2_) (*p* = 0.149) among the groups were found, indicating that most baseline functional and oxygenation indicators were generally comparable across groups (Table 1).

### 3.1. Changes in Outcomes in the Control Group

**Figure 2:**
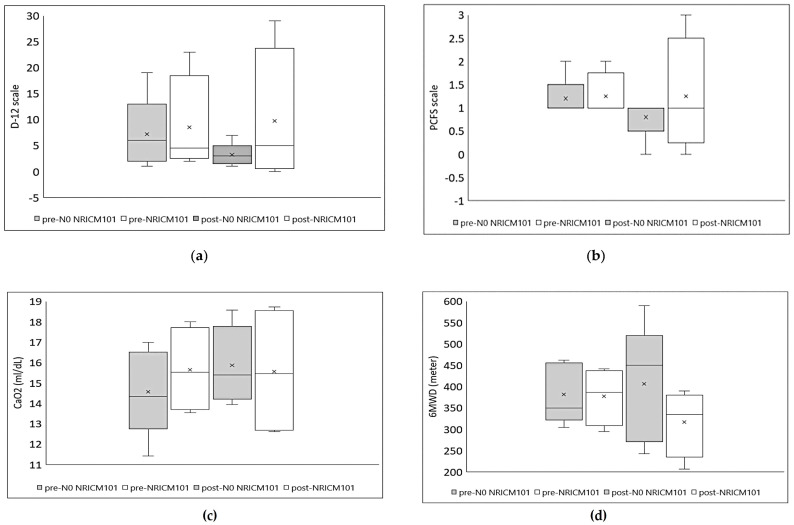
Effects of prior NRICM101 use on (**a**) D-12, (**b**) PCFS, (**c**) CaO_2_, and (**d**) 6MWD outcomes in the control group (CG). D-12 and PCFS are presented as scores. CaO_2_ is expressed in mL/dL. 6MWD is expressed in meters. The box boundaries represent the 25th and 75th percentiles. The horizontal line in the center of the box represents the median. The cross indicates the mean.

Among participants in the control group (CG), no consistent improvement trend was observed in dyspnea severity, functional status, exercise endurance, or CaO_2_ regardless of prior NRICM101 use. In participants without prior NRICM101 use, D-12 scores decreased slightly from 7.2 ± 7.01 to 3.2 ± 2.28, while PCFS scores changed from 1.2 ± 0.44 to 0.8 ± 0.45. Similarly, 6MWD showed only limited variation, ranging from 381.3 ± 70.66 m to 406.1 ± 137.9 m. Participants with prior NRICM101 use also demonstrated limited overall changes in D-12, PCFS, 6MWD, and CaO_2_ values during follow-up. The distributions of post-test outcomes largely overlapped between groups, and no clinically meaningful changes in CaO_2_ were observed. Overall, these findings suggest that without respiratory training intervention, respiratory symptoms, functional status, and exercise tolerance showed limited natural improvement over time (Figure 2).

### 3.2. Changes in Outcomes Among Participants Receiving IS Intervention Within Three Months After Recovery

Among participants who underwent IS intervention within three months after recovery (EG1), both participants with and without prior NRICM101 use showed improvement trends in dyspnea severity and functional status following IS intervention. In participants without prior NRICM101 use, D-12 scores decreased from 9.13 ± 8.02 to 2.4 ± 4.27, while PCFS scores improved from 1.5 ± 0.63 to 0.7 ± 0.6. Similarly, participants with prior NRICM101 use showed decreased D-12 scores (7 ± 4.20 to 1.5 ± 2.51) and improved PCFS scores (1.5 ± 0.55 to 0.5 ± 0.84). The distributions of post-test D-12 and PCFS scores largely overlapped between groups.

**Figure 3:**
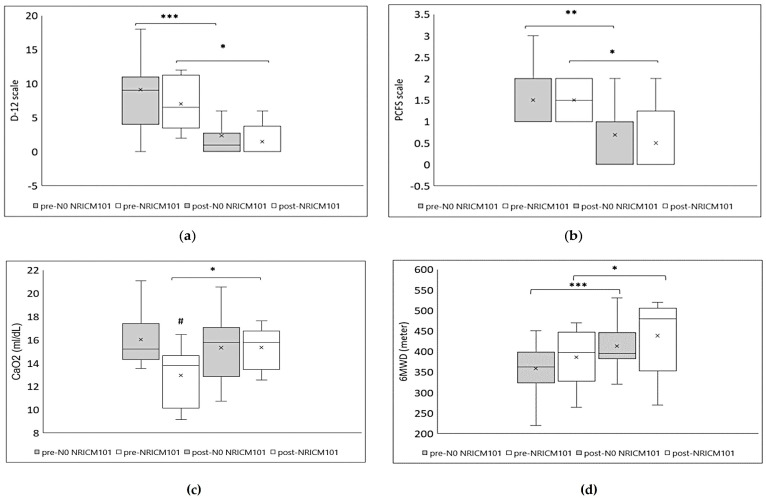
Effects of prior NRICM101 use on outcomes before and after IS intervention in the EG1 group: (**a**) D-12, (**b**) PCFS, (**c**) CaO_2_, and (**d**) 6MWD. D-12 and PCFS are presented as scores. CaO_2_ is expressed in mL/dL. 6MWD is expressed in meters. * *p* < 0.05 vs. baseline; ** *p* < 0.01 vs. baseline; *** *p* < 0.001 vs. baseline. # *p* < 0.05 for comparisons between NRICM101 and no NRICM101 groups. The box boundaries represent the 25th and 75th percentiles. The horizontal line in the center of the box represents the median. The cross indicates the mean.

For exercise endurance, 6MWD increased from 358.7 ± 58.36 m to 413 ± 54.9 m among participants without prior NRICM101 use and from 386.3 ± 73.54 m to 438.3 ± 96.42 m among those with prior NRICM101 use, suggesting an overall improvement trend following respiratory training. In contrast, changes in CaO_2_ were limited overall. Although participants with prior NRICM101 use showed lower baseline CaO_2_ values (13.0 ± 1.09 mL/dL), post-test distributions were similar between groups. Overall, participants in EG1 demonstrated improvement trends in dyspnea, functional status, and exercise endurance following IS intervention regardless of prior NRICM101 use (Figure 3).

### 3.3. Changes in Outcomes Among Participants Receiving IS Intervention Between Three Months and One Year After Recovery

Among participants who received IS intervention between three months and one year after recovery (EG2), both participants with and without prior NRICM101 use showed improvement trends in dyspnea severity and functional status following respiratory training. In participants without prior NRICM101 use, D-12 scores decreased from 6 ± 8.16 to 3.2 ± 6.61, while PCFS scores improved from 1.6 ± 0.89 to 0.6 ± 0.89. Similarly, participants with prior NRICM101 use showed decreased D-12 scores (8.31 ± 6.17 to 2.7 ± 3.35) and improved PCFS scores (1.8 ± 0.44 to 0.7 ± 0.48). The distributions of post-test D-12 and PCFS scores largely overlapped between groups.

**Figure 4:**
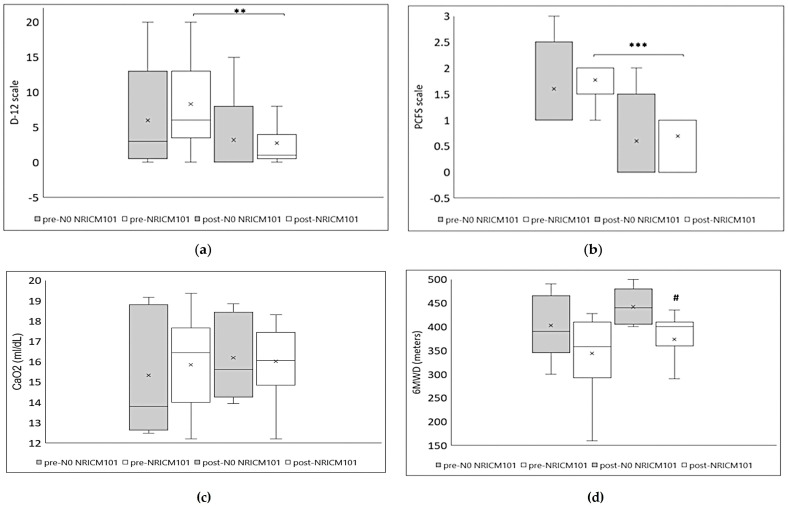
Effects of prior NRICM101 use on outcomes before and after incentive spirometer (IS) intervention in the EG2 group: (**a**) D-12, (**b**) PCFS, (**c**) CaO_2_, and (**d**) 6MWD. D-12 and PCFS are presented as scores. CaO_2_ is expressed in mL/dL. 6MWD is expressed in meters. ** *p* < 0.01 vs. baseline; *** *p* < 0.001 vs. baseline. # *p* < 0.05 for comparisons between NRICM101 and no NRICM101 groups. The box boundaries represent the 25th and 75th percentiles. The horizontal line in the center of the box represents the median. The cross indicates the mean.

For exercise endurance, 6MWD increased from 402.3 ± 70.71 m to 442 ± 40.25 m among participants without prior NRICM101 use and from 343.7 ± 75.37 m to 373.5 ± 57.02 m among those with prior NRICM101 use, indicating a general improvement trend following respiratory training. Although post-test 6MWD distributions differed slightly between groups, overall trends remained similar. In contrast, changes in CaO_2_ were limited in both groups. Participants without prior NRICM101 use showed CaO_2_ values ranging from 15.3 ± 3.22 to 16.2 ± 2.14 mL/dL, while those with prior NRICM101 use showed values ranging from 15.9 ± 0.62 to 16.0 ± 1.83 mL/dL. Overall, among participants who underwent respiratory training after recovery, variations in CaO_2_ remained limited regardless of prior NRICM101 use (Figure 4).

### 3.4. Group-by-Time Interaction Analysis Using Generalized Estimating Equations

The primary inferential analyses of intervention effects were performed using generalized estimating equations (GEE) with group × time interaction models. The CG was used as the reference group to evaluate whether changes between pre-test and post-test outcomes differed between the IS intervention subgroup without prior NRICM101 use (No NRICM101 EG) and the subgroup with prior NRICM101 use (NRICM101 EG).

- Dyspnea index

The GEE analysis revealed a significant difference in the time × group interaction between the two EG groups and the CG, suggesting that the IS intervention led to a more significant decrease in the D-12 score, indicating a greater improvement in symptoms. In particular, the coefficient of time multiplied by group for the NRICM101 EG and No NRICM101 EG was B = −5.526 (95% CI: −7.745 to −3.307, *p* < 0.0001) and B = −5.810 (95% CI: −7.874 to −3.745, *p* < 0.0001), respectively. In contrast, the coefficient of time multiplied by group for the CG was not significant (B = −1.667, *p* = 0.417), showing only natural fluctuations with no consistent trend of improvement (Table 2).

- PCFS functional status

The trend observed for the PCFS score was the same as that for the dyspnea index. In particular, there was a significant difference in the time × group interaction between the two EG groups and the CG, indicating that the IS intervention led to a greater decline in the PCFS score and more significant improvement in functional limitation. As shown in Table 3, the coefficient of time multiplied by group for the NRICM101 EG and No NRICM101 EG was B = −1.053 (95% CI: −1.361 to −0.744, *p* < 0.0001) and B = −0.857 (95% CI: −1.161 to −0.554, *p* < 0.0001), respectively. In contrast, the coefficient of time multiplied by group for the CG was not significant (B = −0.222, *p* = 0.289) (Table 3).

- Exercise endurance

In terms of the 6MWD, both EGs exhibited a significant positive effect on the time × group interaction, showing that the IS intervention led to a greater improvement in the 6MWD score. As shown in Table 4, the coefficient of time multiplied by group for the NRICM101 EG and No NRICM101 EG was B = 36.784 (95% CI: 14.037–59.531, *p* = 0.002) and B = 50.857 (95% CI: 32.931–68.783, *p* < 0.0001), respectively. In contrast, the coefficient of time multiplied by group for the CG was not significant (B = −13.122, *p* = 0.689). Overall, the two EGs showed a marked improvement in exercise tolerance, while no significant changes were observed in the CG (Table 4).

- Arterial oxygen content (CaO_2_)

As summarized in Table 5, in terms of CaO_2_, the NRICM101 EG displayed a significant time × group interaction, with a coefficient of B = 0.875 (95% CI: 0.033–1.718, *p* = 0.042), indicating that the IS intervention led to a greater increase in CaO_2_ and more significant improvement in oxygenation function. In comparison, the coefficient of time multiplied by group was not significant for both the No NRICM101 EG (B = −0.324, *p* = 0.527) and CG (B = 0.685, *p* = 0.129). These results suggest that the interaction between the use of NRICM101 and the IS intervention may be more pronounced in terms of oxygenation parameters. In this regard, the instability of the estimates due to sample size and baseline differences should still be considered (Table 5).

## 4. Discussion

In this study, IS-based respiratory training was used as the primary intervention, and NRICM101 functioned as a prior exposure and comorbidity management rather than a variable of research manipulation. Instead of evaluating the efficacy of NRICM101 itself, this study aimed to investigate whether it may serve as an auxiliary factor that enhances the effectiveness of respiratory rehabilitation.

Pulmonary rehabilitation has been shown to alleviate dyspnea, improve exercise tolerance, and enhance quality of life. The underlying mechanism of action of this intervention may be related to promoting chest expansion, respiratory muscle extension, and improved lung compliance through deep breathing exercises, thereby establishing a more efficient breathing pattern and strengthening respiratory muscles [12]. Randomized controlled trials have shown that inspiratory muscle training (IMT) can improve dyspnea, exercise capacity, and health-related quality of life compared with routine care. Exercise training can also improve the strength of respiratory muscles and the surrounding muscles, functional status, and quality of life. However, high-intensity systemic exercise may be challenging for patients with long COVID who experience persistent fatigue, have limited lung capacity, or are prone to exercise-induced hypoxia, and graded exercise training may not be suitable for all ethnic groups [35,36]. Respiratory training is safer and more adjustable than high-intensity aerobic training and focuses on the function of inspiratory muscles. Thus, it may play a more critical role in rehabilitation strategies for long COVID.

NRICM101 has been reported to primarily act on the inflammatory response and immune regulation during the acute phase of COVID-19 [37]. Its effects theoretically manifest mainly in acute inflammatory responses and subsequent disease recovery trajectories. In contrast, an IS mainly improves lung function and respiratory control by promoting deep inspiration, lung expansion, and respiratory muscle training [12]. Previous studies have demonstrated that IMT can indeed improve dyspnea and functional performance in patients with long COVID [38]. Given that NRICM101 and IS training differ in their underlying mechanisms and intervention timing, these treatments may exhibit different efficacy patterns at different stages of the disease. In this case, if NRICM101 primarily affects inflammatory responses and symptom development in the acute phase, it may indirectly alter the subsequent recovery trajectory rather than directly enhancing the effectiveness of IS-based respiratory training [9,39]. In the present study, participants were divided into two groups for a comparative analysis based on the time after recovery: “within three months” and “ within three months to one year ”. If intergroup differences are still observed more than three months after recovery, this observation may reflect differences in symptom burden, disease course phenotype, or health behavior rather than being entirely attributable to pharmacological effects.

Upon analyzing the interaction between time and group using GEE without considering the timing of IS intervention, we observed a trend toward greater improvement in PCFS functional status among participants who had previously used NRICM101 compared with those who had not used it; however, this finding should be interpreted cautiously given the small subgroup sizes. A stratified analysis incorporating the timing of the intervention revealed that participants who underwent respiratory training within three months after recovery, regardless of whether NRICM101 was administered, showed an improvement in symptoms, and this improvement was slightly greater in those who did not use it.

In contrast, among participants who received the intervention more than three months after recovery, a more pronounced trend of improvement in respiratory and functional status was observed in those with prior NRICM101 use. In practical clinical settings, the use of NRICM101 is often influenced by various factors, such as the timing of initiation, duration of treatment, use of concomitant therapies, and patient compliance. Variations in these factors may lead to instability in the estimation of treatment efficacy [17].

However, the potential impact of health behaviors and treatment adherence should also be considered. Patients who have taken NRICM101 may demonstrate a higher level of treatment engagement or a more proactive tendency toward healthy behaviors, such as a greater willingness to participate in home-based exercises or a higher level of trust in interventions. Previous research has indicated that adherence itself is an important health behavior, and patients with high adherence generally engage more actively in overall health management [40,41]. Therefore, the functional improvements observed in the present study may partly reflect differences in patients’ health behaviors rather than being entirely attributable to the intervention itself. Moreover, higher adherence may have led participants to perform respiratory training more regularly and consistently. This is particularly true in the management of chronic diseases or symptoms, where treatment typically involves multiple non-pharmacological strategies, resulting in a closer association between adherence and efficacy [42].

## 5. Limitation

In the present study, some of the results may also be subject to the ceiling effect or disparities in the potential for improvement, resulting in more pronounced changes in certain indicators under specific stratification. The ceiling effect may reduce the sensitivity of measurement tools to changes. A high level of baseline functioning has been suggested to be correlated with limited observable symptom improvement, while significant improvement is more likely to be observed when baseline symptoms are more severe [43].

The distribution of cardiovascular disease differed significantly between groups and may have influenced exercise tolerance, dyspnea severity, and oxygenation-related outcomes. Due to the limited number of affected participants, further multivariable adjustment was not considered statistically stable in the present exploratory analysis. Objective pulmonary function measurements such as DLco, FEV1, FVC, and respiratory muscle strength were also not assessed in the present study, which may limit the physiological interpretation of respiratory rehabilitation outcomes.

The distribution of cardiovascular disease differed significantly between groups and may have influenced exercise tolerance, dyspnea severity, and oxygenation-related outcomes. Due to the limited number of affected participants, further multivariable adjustment was not considered statistically stable in the present exploratory analysis. Objective pulmonary function measurements such as DLco, FEV1, FVC, and respiratory muscle strength were also not assessed in the present study, which may limit the physiological interpretation of respiratory rehabilitation outcomes. In addition, as this study was conducted during the later stage of the COVID-19 pandemic, the intervention was performed independently in a home-based setting, and repeated in-person follow-up visits were not required in order to minimize face-to-face contact and reduce the potential risk of infection. Therefore, adherence assessment primarily relied on participant self-report and telephone follow-up. Participants received standardized instruction and return demonstration before beginning the intervention; however, objective adherence measures, such as completion rates and missed-session records, were not collected. Furthermore, prior NRICM101 use was self-reported at study entry, and therefore recall bias could not be completely excluded.

Although the findings of the present study reflect treatment choices and patient behaviors in real-world clinical settings, causal inference is limited due to the lack of a randomized design in the subgroup analyses. Future research should more precisely define drug exposure conditions, such as the cumulative days of use, actual dosage, and time of initiation, to assess their potential effects more accurately. Moreover, because the present NRICM101-related analysis was conducted as an exploratory secondary analysis with further subgroup stratification, several subgroup sample sizes were relatively small, which may have limited statistical power and increased estimate instability. Therefore, the findings should be interpreted with caution.

## 6. Conclusions

This study investigated the effects of respiratory training using an IS on dyspnea and functional status in patients with long COVID and further analyzed whether prior use of NRICM101 might influence rehabilitation outcomes and disease recovery. Compared with the CG that did not receive respiratory training, patients who underwent IS training appeared to show improvements in dyspnea, functional status, and exercise tolerance. However, these findings should be interpreted cautiously and may serve as preliminary evidence supporting further investigation of respiratory training in the rehabilitation of long COVID. Further analysis revealed that a trend toward greater improvements was observed among patients who had previously used NRICM101 (e.g., PCFS and CaO_2_) than those who did not use this formula; however, this difference was inconsistent across different stratifications based on the time after recovery (within three months vs. within three months to one year). These findings may reflect differences in disease recovery trajectories rather than a direct enhancement of respiratory training outcomes by NRICM101. Given that the use of NRICM101 in this study was not randomly assigned and that the current research findings may be subject to the influence of treatment timing, duration of treatment, and patient health behaviors, they should be interpreted with caution and considered hypothesis-generating rather than confirmatory evidence of a treatment effect. Future studies could further clarify the potential interaction between traditional medical treatments and rehabilitation interventions by using larger sample sizes and prospective designs and by defining drug exposure conditions more accurately.

## Figures and Tables

**Table 1:** Comparison of baseline sociodemographic and clinical characteristics among groups (*n* = 49).

			No NRICM101			NRICM101			
		All	CG	EG1	EG2	CG	EG1	EG2	*p Value*
		(*n* = 49)	(*n* = 5)	(*n* = 16)	(*n* = 5)	(*n* = 4)	(*n* = 6)	(*n* = 13)	
**Gender [N (%)]**									0.461
	**Male**	20 (40.8%)	0 (0%)	7 (43.8%)	3 (60%)	2 (50%)	3 (50%)	5 (38.5%)	-
	**Female**	29 (59.2%)	5 (100%)	9 (56.3%)	2 (40%)	2 (50%)	3 (50%)	8 (61.5%)	-
**Age [Mean (Median)]**		39.5 (34)	33 (31)	38.9 (33.5)	41 (42)	38.3 (37.5)	40 (40.5)	42.2 (35)	0.88
**Height [Mean (Median)]**		166.3 (167)	161 (160)	166.4 (165.5)	171.2 (170)	163 (163)	169.4 (173.5)	165.7 (163)	0.319
**Weight [Mean (Median)]**		68.8 (67)	60.8 (56)	71.5 (76)	80.6 (80)	62.3 (62)	65.5 (65)	67.7 (69)	0.136
**Duration Post-Recovery** **[Mean (Median)]**		4.6 (3)	3.9 (2)	1.5 (1)	7.9 (8)	7.8 (8.5)	1.2 (1)	7.9 (8)	<0.017 *
**Hypertension [N (%)]**									0.725
	**Yes**	8 (16.3%)	0 (0%)	4 (25%)	1 (20%)	1 (25%)	1 (16.7%)	1 (7.7%)	-
	**No**	41 (49%)	5 (100%)	12 (75%)	4 (80%)	3 (75%)	5 (83.3%)	12 (92.3%)	-
**Cardiovascular Disease [N (%)]**									0.006 *
	**Yes**	4 (8.2%)	0 (0%)	1 (6.3%)	0 (0%)	0 (0%)	3 (50%)	0 (0%)	-
	**No**	45 (91.8%)	5 (100%)	15 (93.8%)	5 (100%)	4 (100%)	3 (50%)	13 (100%)	-
**Diabetes [N (%)]**									0.831
	**Yes**	3 (6.1%)	0 (0%)	1 (6.3%)	0 (0%)	0 (0%)	1 (16.7%)	1 (7.7%)	-
	**No**	46 (93.9%)	5 (100%)	15 (93.8%)	5 (100%)	4 (100%)	5 (83.3%)	12 (92.3%)	-
**Smoking [N (%)]**									0.725
	**Yes**	8 (16.3%)	0 (0%)	4 (25%)	1 (20%)	1 (25%)	1 (16.7%)	1 (7.7%)	-
	**No**	41 (83.7%)	5 (100%)	12 (75%)	4 (80%)	3 (75%)	5 (83.3%)	12 (92.3%)	-
**Number of Infections [N (%)]**									0.148
	**1 time**	26 (53.1%)	1 (20%)	5 (31.3%)	4 (80%)	3 (75%)	5 (83.3%)	8 (61.5%)	-
	**2 times**	20 (40.8%)	4 (80%)	9 (56.3%)	1 (20%)	1 (25%)	0 (0%)	5 (38.5%)	-
	**3 times**	3 (6.1%)	0 (0%)	2 (12.5%)	0 (0%)	0 (0%)	1 (16.7%)	0 (0%)	-
**Exercise [N (%)]**									0.258
	**No**	18 (36.7%)	2 (40%)	8 (50%)	1 (20%)	0 (0%)	1 (16.7%)	6 (46.2%)	-
	**1~3 times/week**	23 (46.9%)	3 (60%)	4 (25%)	2 (40%)	4 (100%)	5 (83.3%)	5 (38.5%)	-
	**4~5 times/week**	4 (8.2%)	0 (0%)	3 (18.8%)	1 (20%)	0 (0%)	0 (0%)	0 (0%)	-
	**6~7 times/week**	4 (8.2%)	0 (0%)	1 (6.3%)	1 (20%)	0 (0%)	0 (0%)	2 (15.4%)	-
**PCFS [Mean (Median)]**		1.53 (1)	1.2 (1)	1.5 (1)	1.6 (1)	1.3 (1)	1.5 (1.5)	1.8 (2)	0.235
**D-12 [Mean (Median)]**		8.1 (6)	7.2 (6)	9.13 (9)	6 (3)	8.5 (4.5)	7 (6.5)	8.31 (6)	0.604
**6MWD (meter) [Mean (Median)]**		366.4 (366)	381.3 (350)	358.7 (363)	402.3 (390)	377.3 (386.3)	386.33 (397)	343.7 (358)	0.572
**Hb (g/dL) [Mean (Median)]**		11.7 (11.6)	11 (10.8)	12.1 (11.75)	12 (10.5)	11.9 (11.9)	9.8 (10.4)	12 (12.4)	0.122
**Hct (%) [Mean (Median)]**		35.1 (34.8)	33 (32.5)	36.4 (35.4)	36 (32)	35.9 (35.8)	29.4 (31.25)	36.2 (37.2)	0.115
**SpO_2_ (%) [Mean (Median)]**		98.6 (99)	99 (99)	98.8 (99)	98.4 (99)	98.3 (99)	98.7 (99)	98.5 (99)	0.204
**CaO_2_ (mL/dL) [Mean (Median)]**		15.3 (15.15)	14.6 (14.33)	16 (15.22)	15.3 (13.8)	15.6 (15.52)	13 (13.8)	15.8 (16.5)	0.149

Note 1: PCFS = Post-COVID-19 Functional Status total score; D-12 = Dyspnoea-12 total score; 6MWD = Six-Minute Walk Distance. Note 2: * = *p* < 0.05.

**Table 2:** GEE analysis of group-by-time interaction effects on D-12.

	B	SE	95% Wald CI		Hypothesis Test		
			Lower	Upper	Wald χ^2^	Df	*p*-Value
**NRICM101 EG × time**	−5.526	1.1322	−7.745	−3.307	23.826	1	*p* < 0.0001 ***
**No NRICM101 EG × time**	−5.810	1.0533	−7.874	−3.745	30.423	1	*p* < 0.0001 ***
**CG × time**	−1.667	2.0548	−5.694	2.361	0.658	1	0.417
**NRICM101 EG**	3.977	4.1970	−4.249	12.203	0.898	1	0.343
**No NRICM101 EG**	4.746	4.4061	−3.890	13.382	1.160	1	0.281
**CG**	0a						
**time**	0a						

Note: *** = *p* < 0.001.

**Table 3:** GEE analysis of group-by-time interaction effects on PCFS.

	B	SE	95% Wald CI		Hypothesis Test		
			Lower	Upper	Wald χ^2^	Df	*p*-Value
**NRICM101 EG × time**	−1.053	0.1574	−1.361	−0.744	44.706	1	*p* < 0.0001 ***
**No NRICM101 EG × time**	−0.857	0.1548	−1.161	−0.554	30.649	1	*p* < 0.0001 ***
**CG × time**	−0.222	0.2095	−0.633	0.188	1.125	1	0.289
**NRICM101 EG**	1.292	0.3266	0.652	1.932	15.661	1	*p* < 0.0001 ***
**No NRICM101 EG**	0.937	0.3497	0.251	1.622	7.171	1	0.007 **
**CG**	0a						
**time**	0a						

Note: ** = *p* < 0.005; *** = *p* < 0.001.

**Table 4:** GEE analysis of group-by-time interaction effects on 6MWD.

	B	SE	95% Wald CI		Hypothesis Test		
			Lower	Upper	Wald χ^2^	Df	*p*-Value
**NRICM101 EG × time**	36.784	11.6058	14.037	59.531	10.046	1	0.002 **
**No NRICM101 EG × time**	50.857	9.1461	32.931	68.783	30.919	1	*p* < 0.0001 ***
**CG × time**	−13.122	32.8400	−77.487	51.243	0.160	1	0.689
**EG2**	−72.254	46.4887	−163.371	18.862	2.416	1	0.120
**EG1**	−74.438	44.7871	−162.219	13.343	2.762	1	0.097
**CG**	0a						
**time**	0a						

Note: ** = *p* < 0.005; *** = *p* < 0.001.

**Table 5:** GEE analysis of group-by-time interaction effects on CaO_2_.

	B	SE	95% Wald CI		Hypothesis Test		
			Lower	Upper	Wald χ^2^	Df	*p*-Value
**NRICM101 EG × time**	0.875	0.4300	0.033	1.718	4.145	1	0.042 *
**No NRICM101 EG × time**	−0.324	0.5122	−1.328	0.680	0.400	1	0.527
**CG × time**	0.685	0.4515	−0.200	1.569	2.299	1	0.129
**EG2**	−0.302	1.2665	−2.784	2.181	0.057	1	0.812
**EG1**	1.816	1.1986	−0.533	4.165	2.296	1	0.130
**CG**	0a						
**time**	0a						

Note: * = *p* < 0.05.

## Data Availability

The pulmonary function test data and clinical information in the current study are not publicly available due to patient privacy obligations but are available from the corresponding author upon reasonable request.

## Abbreviations

The following abbreviations are used in this manuscript:

COVID-19	Coronavirus disease 2019
IS	Incentive Spirometer
SARS	Severe acute respiratory syndrome
MERS	Middle East respiratory syndrome
SARS-CoV-2	Severe acute respiratory syndrome coronavirus
WHO	World Health Organization
ERS	European Respiratory Society
ATS	American Thoracic Society
NRICM101	Taiwan Chingguan Yihau
RT-PCR	Reverse transcription polymerase chain reaction
HIF-1	Hypoxia-inducible factor 1
TNF	Tumor necrosis factor
D-12	Dyspnoea-12
PCFS	Post-COVID-19 Functional Status
6MWT	Six-Minute Walk Test
6MWD	Six-Minute Walk Distance
GEEs	Generalized Estimating Equations
Hb	Hemoglobin
Hct	Hematocrit
SpO_2_	Peripheral oxygen saturation
CaO_2_	Arterial oxygen content
